# Divanillin Cross‐Linked Recyclable Cellulose Networks

**DOI:** 10.1002/marc.202401094

**Published:** 2025-03-26

**Authors:** Meiling Zhang, Sathiyaraj Subramaniyan, Minna Hakkarainen

**Affiliations:** ^1^ KTH Royal Institute of Technology Department of Fibre and Polymer Technology Stockholm 10044 Sweden; ^2^ College of Textile Engineering Taiyuan University of Technology Jinzhong Shanxi 030600 China; ^3^ KTH Royal Institute of Technology Wallenberg Wood Science Center (WWSC) Stockholm 10044 Sweden

**Keywords:** cellulose, circular materials, covalent adaptable networks, recycling, vanillin

## Abstract

A series of cellulose networks are designed by reversibly crosslinking amino‐functionalized 2‐hydroxyethyl cellulose (HEC‐NH_2_) with different amounts of vanillin dimer (VA‐CHO). The Schiff base reaction between amino‐and aldehyde groups creates networks (SBHEC) bridged with crosslinks containing dynamic imine groups. These SBHEC networks can be hot pressed to flexible films with good thermal stability and solvent resistance, including notable stability in water, opposite to water‐soluble HEC and HEC‐NH_2_. Compared to HEC‐NH_2_, the cross‐linked SBHEC networks exhibit higher glass transition temperatures, elastic modulus, and tensile stress at break, and slightly reduced tensile strain at break. Reprocessing of the SBHEC networks is achieved through hot pressing under facile conditions, leading to good recovery of mechanical properties. Furthermore, the materials can be chemically recycled in a closed‐loop by imine‐hydrolysis under acidic conditions at room temperature. This releases the original building blocks HEC‐NH_2_ and VA‐CHO, which can be recured to produce new SBHEC. This work highlights the potential of dynamic covalent cellulose networks as mechanically and chemically recyclable materials, contributing to the development of closed‐loop recycling systems.

## Graphical abstract

Dynamic cellulose networks are designed through a Schiff base reaction between amino‐functionalized 2‐hydroxyethyl cellulose and vanillin dimer. The obtained materials can be hot‐pressed to flexible films with good thermal stability and solvent resistance. Furthermore, the films are mechanically and chemically recyclable in closed‐loop.

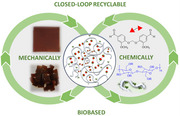

## 1 Introduction

The combination of durability, lightness, cost‐effectiveness, and customizability make polymers a popular choice for use in many products in our daily lives. Currently, most polymers are prepared using fossil‐based feedstocks and less than 20% are recycled after use.^[^ ^1^, ^2^ ^]^ Notably, the current thermosets and elastomers are to an even higher degree derived from nonrenewable fossil‐based feedstocks and they are difficult to degrade or recycle after service owing to the permanent cross‐linked structures and often high aromatic contents.^[^ ^3^, ^4^ ^]^ At the same time, the irreversible cross‐linked network provides these materials with enhanced material properties.^[^ ^5^ ^]^ To address these issues, new and innovative ways are explored to design more sustainable polymers originating from renewable resources and adjusted to circular material flows.^[^ ^6^, ^7^, ^8^ ^]^

Dynamic covalent chemistry (DCC) was first introduced by WUDL and co‐workers.^[^ ^9^ ^]^ The term covalent adaptable networks (CAN) was coined by Bowman^[^ ^10^ ^]^ and vitrimer by Leibler^[^ ^11^ ^]^ both referring to cross‐linked polymer networks exhibiting reversible bonds. The DCC chemistry ideally enables the excellent properties of covalently cross‐linked polymers,^[^ ^12^ ^]^ at the same time as the materials, unlike traditional cross‐linked polymers, can be melted and reshaped without losing the properties and structure.^[^ ^13^, ^14^, ^15^ ^]^ The reversibly cross‐linked materials have great potential in the development of circular polymer materials, due to the wide availability of chemistries, such as Schiff base,^[^ ^16^ ^]^ Diels‐Alder,^[^ ^17^ ^]^ disulfide exchange^[^ ^18^ ^]^ and transesterification.^[^ ^19^, ^20^ ^]^ Among the recognized dynamic bonds, the imine bond can undergo both associative (amine exchange) and dissociative (imine hydrolysis and reformation) reactions.^[^ ^21^ ^]^ Furthermore, many bio‐based aldehydes can be utilized for Schiff base reactions.

Cellulose, the most abundant natural polymer, has gained significant attention due to its interesting properties, biocompatibility, and biodegradability.^[^ ^22^, ^23^ ^]^ Cellulose and its derivatives are also envisaged as promising raw materials for bio‐based CAN, because of the numerous reactive groups^[^ ^24^ ^]^ and the prevalence of biobased monomers, such as hydroxy acids, amines, and aldehydes, that offer almost unlimited potential to functionalize cellulose and to redesign plastics that are chemically and mechanically recyclable and/or biodegradable.^[^ ^25^, ^26^ ^]^ Vanillin is an interesting sustainable chemical derivable from lignin. It contains a rigid benzene ring and an aldehyde group, which can be utilized to design Schiff base vitrimers.^[^ ^16^, ^27^, ^28^ ^]^ Over the last decade, many permanently cross–linked thermosets were derived from bio‐based resources^[^ ^29^, ^30^ ^]^ while the focus was more recently shifted to bio‐based dynamic networks^[^ ^31^ ^]^ to enable e.g., self‐healable materials and closed‐loop recycling.^[^ ^32^ ^]^

Many commercial thermoplastic cellulose derivatives, such as 2‐hydroxyethyl cellulose (HEC), are water‐soluble and brittle limiting the application range.^[^ ^33^, ^34^ ^]^ We hypothesized that the introduction of reversible crosslinks between cellulose chains could help to overcome these limitations and facilitate the production of flexible cellulose products with increased stability in water. Furthermore, the reversibility of the crosslinks would still enable thermal reprocessing and provide a route to chemical recyclability under mild conditions to fully integrate these biobased materials into a circular material economy. Therefore, we designed HEC‐derived CAN by Schiff base reaction between amino‐modified 2‐hydroxyethyl cellulose (HEC‐NH_2_) and biobased vanillin dimer (VA‐CHO) crosslinker aiming at a combination of thermal stability, solvent resistance, and closed‐loop recyclability.

## 2 Results and Discussion

### 2.1 Synthesis and Characterization of SBHEC

**Scheme 1:**
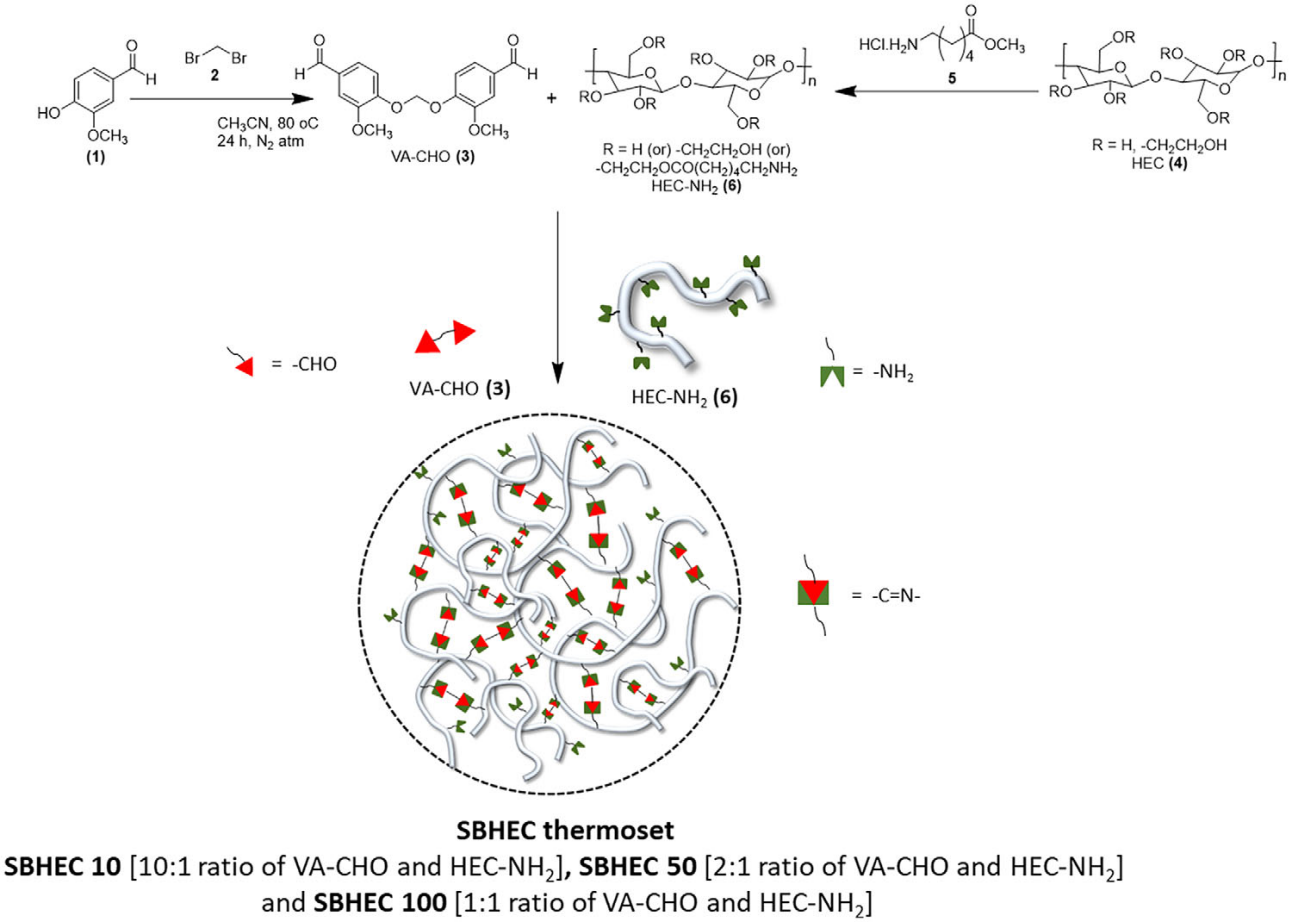
Schematic reaction scheme for the synthesis of SBHEC 50 and SBHEC 100.

Cellulose‐derived dynamic networks integrating attractive thermomechanical properties with chemical and mechanical recyclability were designed. The SBHEC were fabricated according to the following thesis: i) the commercial cellulose derivative HEC was functionalized with amine groups; ii) vanillin‐dimer with two aldehyde groups was synthesis as a bio‐based cross‐linker; iii) the dynamic network was formed by Schiff base reaction between the amine‐groups in the functionalized HEC chains and the aldehyde groups of the vanillin dimers (**Scheme** **1**).

**Figure 1:**
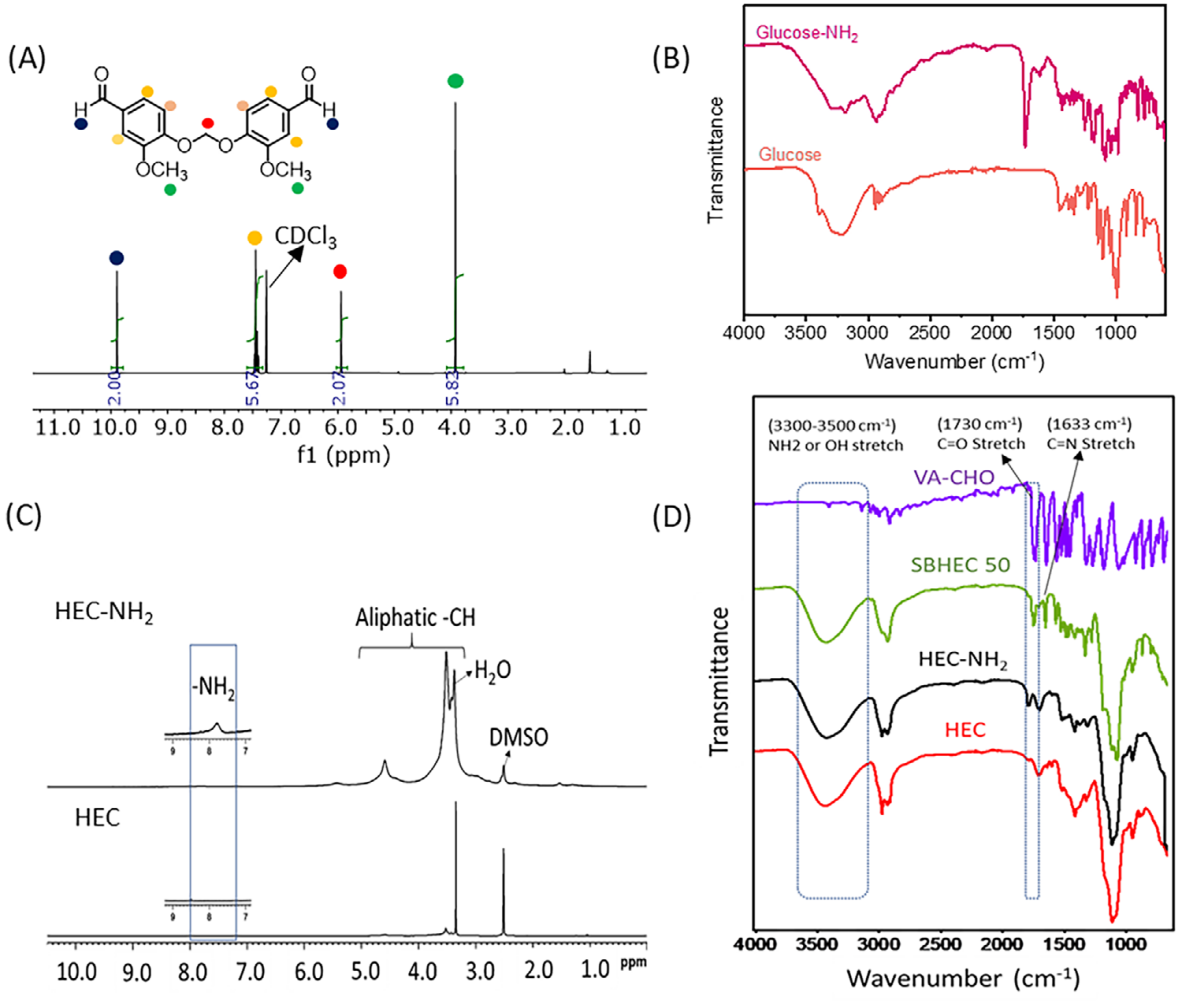
Design and synthesis of VA‐CHO, HEC‐NH_2_, and SBHEC. A) ^1^H NMR spectrum of VA‐CHO. B) FTIR curves of glucose and amine‐functionalize glucose. C) ^1^H NMR spectrum of HEC and HEC‐NH_2_. D) FTIR spectra of VA‐CHO, HEC, HEC‐NH_2_, and SBHEC.

The VA‐CHO cross‐linker was first synthesized by reacting vanillin with dibromomethane and the reaction was confirmed by ^1^H NMR (**Figure** **1**A). The aldehyde hydrogen was observed at δ ≈9.80 ppm and remaining hydrogens including aromatic ─CH, ─OCH_2,_ and ─OCH_3_ appeared at ≈ δ 7.50, 5.90, and 3.80 ppm, respectively. In the FTIR spectra of VA‐CHO (Figure 1D), the bands at 1602, 1574 and 1725 cm^−1^ are attributed to the aldehyde group. HEC‐NH_2_ was fabricated by a reaction between HEC and methyl 6‐aminohexanoate hydrochloride. To verify the reaction, a model experiment, i.e., a reaction between glucose and methyl 6‐aminohexanoate hydrochloride, was first conducted. ^1^H NMR and FTIR spectra of the amino‐modified glucose confirmed the successful model reaction (Figure 1B; Figure S1, Supporting Information). This is depicted for example by the appearance of a strong carbonyl‐ester absorption band at 1730 cm^−1^ demonstrating the successful reaction between hydroxyl‐groups of glucose and methyl 6‐aminohexanoate hydrochloride. After verification of the successful model reaction, HEC was reacted with methyl 6‐aminohexanoate hydrochloride, and the chemical structure of the resulting HEC‐NH_2_ was investigated by ^1^H NMR (Figure 1C). The successful reaction was confirmed by the emergence of an amino‐group signal at 7.75 ppm in the NMR spectrum. As further shown by the FTIR spectra in Figure 1D, the new peak at ≈1730 cm^−1^ corresponding to ester carbonyl (─C ═O) appeared after the reaction. In the next step, HEC‐NH_2_ was cross‐linked by Schiff base reaction with VA‐CHO. A successful reaction was supported by the appearance of a band at ≈1633 cm^−1^ indicating the formation of imine (C═N) bonds.

**Figure 2:**
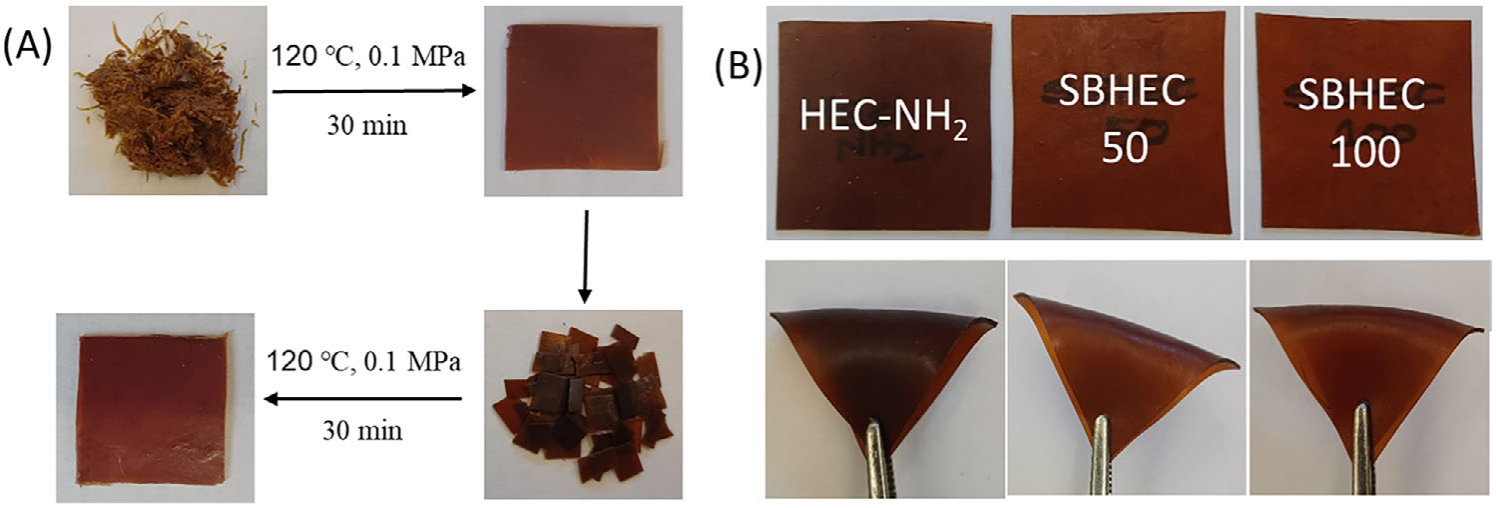
Digital photos showing A) original material, a hot‐pressed film, and the reprocessing cycle including cutting into pieces followed by a second hot pressing and B) the flexible HEC‐NH_2_, SBHEC 50, and SBHEC 100 films.

Films of HEC, HEC‐NH_2_, SBHEC 50, and SBHEC 100 were prepared using hot pressing (**Figure** **2**). The hot‐pressed films were then subjected to analysis of thermal and mechanical properties before they were cut into pieces and hot‐pressed for a second time followed again by analysis of the thermal and mechanical properties to evaluate the reprocessability (Figure 2).

### 2.2 Thermal Properties and Chemical Structure Before and After Reprocessing

**Figure 3:**
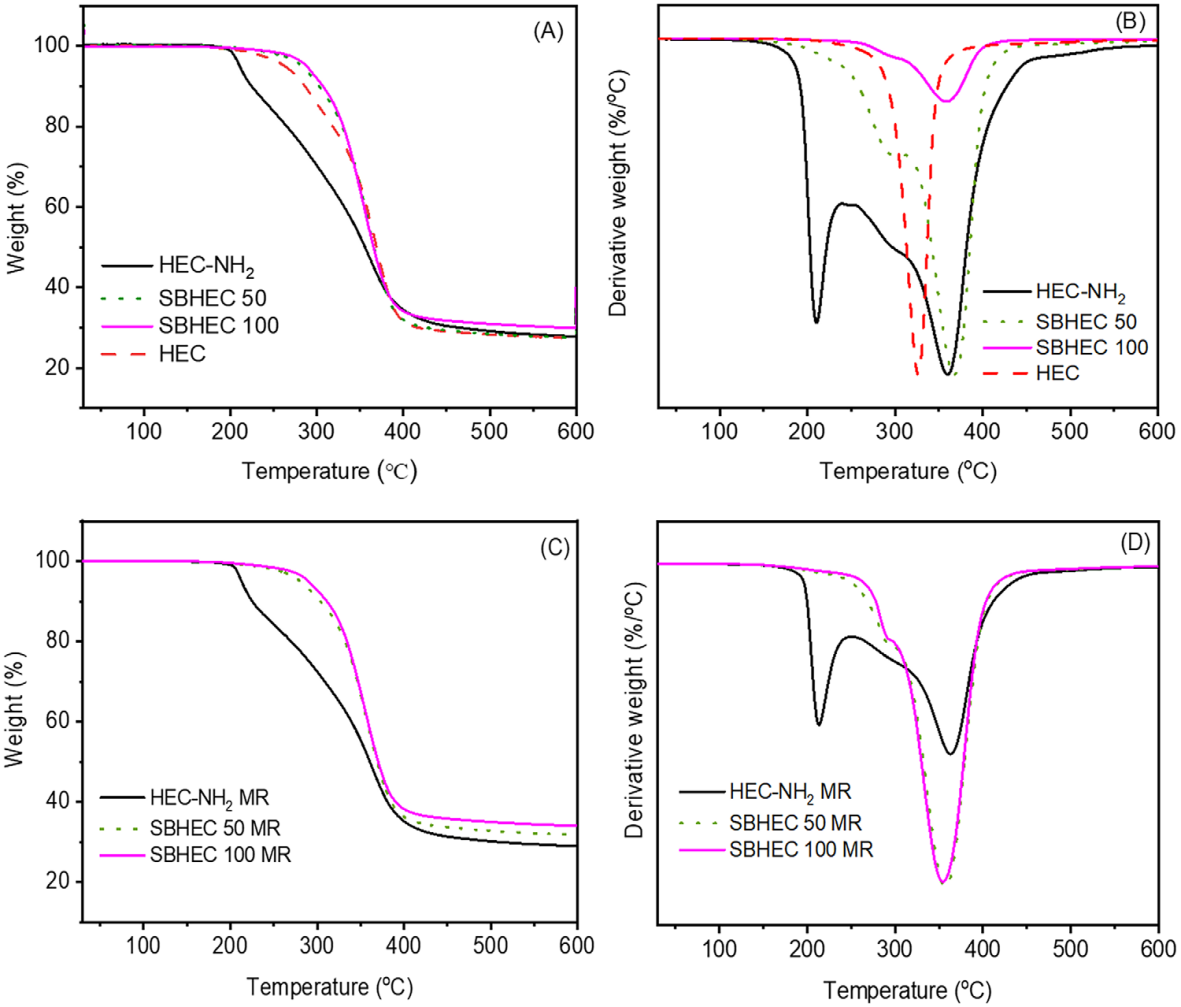
A) TGA and B) DTG curves of HEC, HEC‐NH2, SBHEC 50 and SBHEC 100. C) TGA and D) DTG curves of reprocessed HEC‐NH_2_ MR, SBHEC 50 MR, and SBHEC 100 MR.

The thermal stability of HEC, VA‐CHO, HEC‐NH_2_, SBHEC 50, and SBHEC 100 was evaluated by using TGA. The TGA and DTG curves of the original and reprocessed films are shown in **Figures** **3** and S2 (Supporting Information) and the results are summarized in **Table** **1**. In comparison with all the other materials HEC‐NH_2_ exhibited lower thermal stability and a prominent two‐step or possibly three‐step degradation process, which was likely initiated by the free amino groups (Figure 3A,B). The degradation onset temperature of HEC‐NH_2_ as determined by 5% weight loss (*T_5_*) was observed already at 210 °C and the two decomposition maxima (*T_d_*) at 213 and 360 °C. This multistep process and lower thermal stability were also observed for the reprocessed HEC‐NH_2_ MR films, which exhibited similar *T_5_* and *T_d_* values to the original films (Figure 3C,D). Both SBHEC materials exhibited two‐step degradation mechanisms, however, the first step was much less prominent and appeared at higher temperatures compared to HEC‐NH_2._ Similar two‐step decomposition mechanisms were previously observed for the Schiff base network, where dialdehyde cellulose nanofibrils were cross‐linked with diamines.^[^ ^35^ ^]^ *T_5_* temperatures 282 and 289 °C were observed for the original SBHEC 50 and SBHEC 100 films, which is ≈70–80 °C higher compared to HEC‐NH_2_ (Figure 3C,D). These temperatures are intermediate to what was previously reported for vanillin‐derived Schiff base networks, which typically have *T_5_* values from 250 °C (polyester‐imines)^[^ ^27^ ^]^ to 320 °C (polyimine‐amides).^[^ ^36^ ^]^ The *T_d_* temperature for the first minor decomposition step was 295 °C, which is also ≈80 °C higher compared to HEC‐NH_2_. The *T_d_* temperatures for the main decomposition step were in the range of 363–369 °C, which is similar to HEC‐NH_2_ and somewhat higher than the *T_d_* (327 °C) of the original HEC. The crosslinking by Schiff base reaction with VA‐CHO, thus, significantly increased the thermal stability of the materials, which supports the formation of cross‐linked structures via Schiff base reaction.^[^ ^37^ ^]^ Moreover, the reprocessing did not significantly influence the thermal stability.

**Table 1:** Thermal and mechanical properties of HEC, VA‐CHO, HEC‐NH_2_, SBHEC 50, and SBHEC 100 before and after reprocessing.

Samples	TGA		T_g_ [°C] [DMA]	Elastic modulus [MPa]	Tensile strain at break [%]	Tensile stress at break [MPa]
	*T_5_* [°C]	*T_d_* [°C]				
HEC	288	327	118	4524 ± 398	9 ± 1.4	157 ± 21.5
HEC MR	289	329	121	3265 ± 126	8 ± 1.1	118 ± 7.0
VA‐CHO	280	346	–	–	–	–
HEC‐NH_2_	210	213, 360	65	12.6 ± 11.1	28 ± 7.8	1.7 ± 0.2
HEC‐NH_2_ MR	213	213, 363	66	14.8 ± 3.8	19 ± 3.7	0.8 ± 0.3
SBHEC 50	282	295, 369	94	19.9 ± 9.3	24 ± 5.4	2.9 ± 1.4
SBHEC 50 MR	281	293, 354	98	27.5 ± 6.0	21 ± 1.8	3.2 ± 0.8
SBHEC 100	289	295, 363	98	36.3 ± 8.0	23 ± 4.4	3.3 ± 0.4
SBHEC 100 MR	290	295, 355	99	28.4 ± 3.3	23 ± 4.8	2.8 ± 0.4

**Figure 4:**
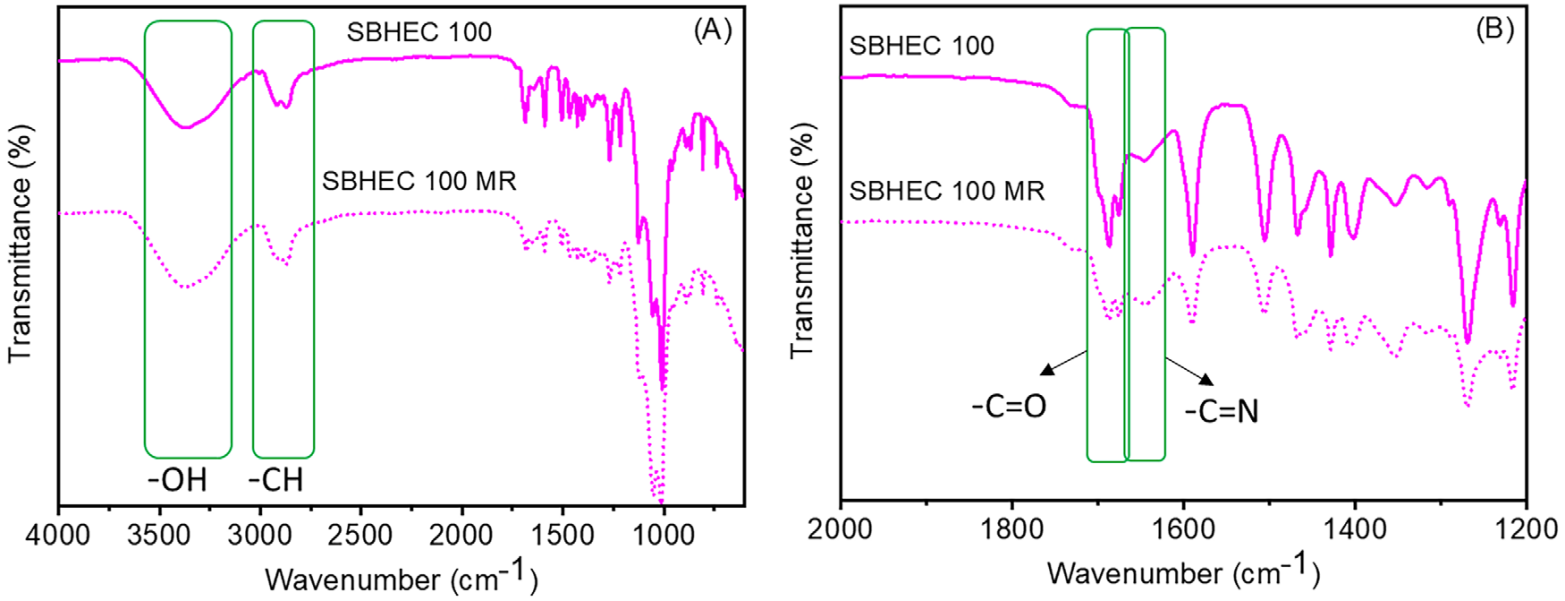
FTIR spectra of original and mechanically recycled SBHEC 100.

**Figure 5:**
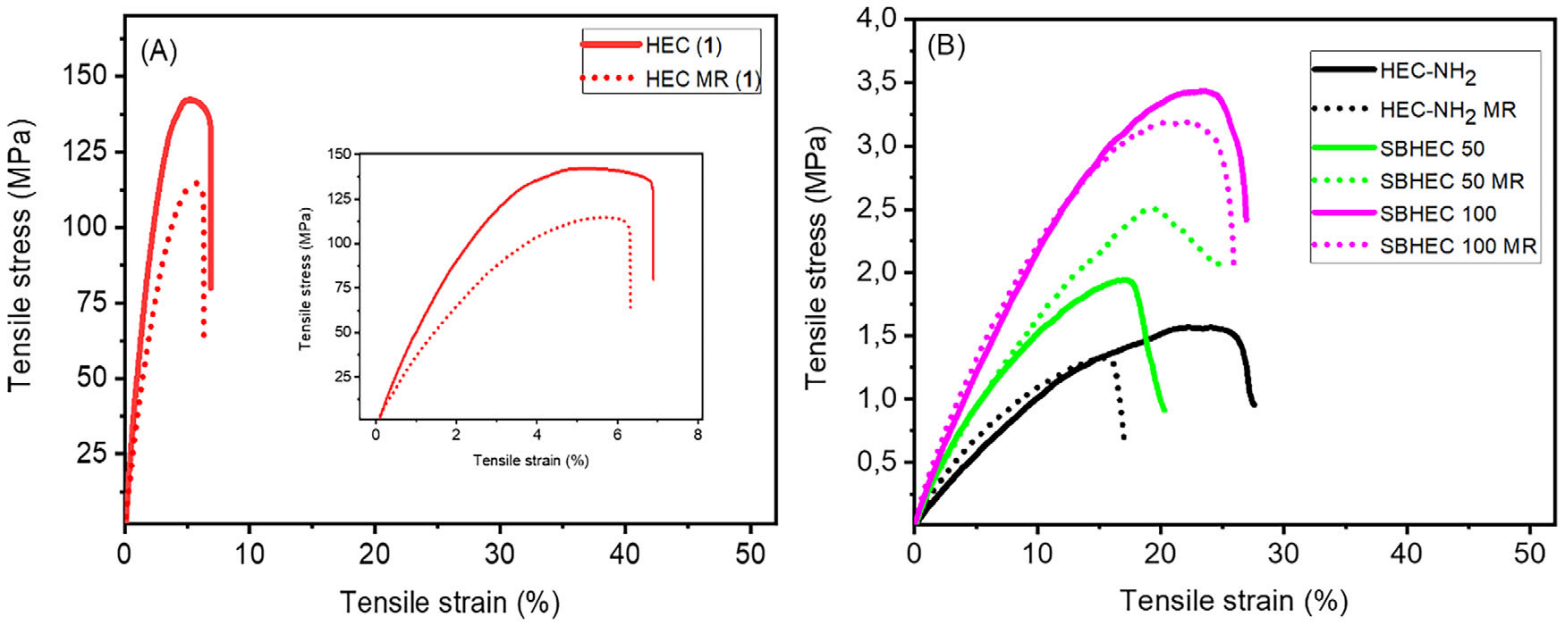
Stress–strain curves of original and reprocessed samples A) HEC and B) HEC‐NH_2_, SBHEC 50 and SBHEC 100.

After the reprocessing, the chemical structures of HEC MR, HEC‐NH_2_ MR, SBHEC 50 MR, and SBHEC 100 MR were confirmed by FTIR analysis. No major changes in the spectra were observed. The FTIR spectra of original and recycled SBHEC 100 are shown as examples in **Figure** **4**, while the remaining spectra can be found in supporting information, Figure S3 (Supporting Information). *T_g_* as determined from *tan δ* of original and reprocessed films was investigated by DMA, Table 1. The original HEC was a brittle and stiff material with relatively high *T_g_*. The modification of HEC with methyl‐6‐aminohexanoate to form HEC‐NH_2_, decreased the *T_g_* by ≈50 °C and increased the flexibility of the material (See Figures 2 and **5**). The cross‐linking of HEC‐NH_2_ chains by Schiff base reaction with the dialdehyde, VA‐CHO to form SBHEC50 and SBHEC 100 led to ≈30 °C increase in *T_g_*. No major changes in *T_g_* values were observed after recycling.

### 2.3 Tensile Properties Before and After Reprocessing

The mechanical properties of original and reprocessed HEC, HEC‐NH_2_, SBHEC 50, and SBHEC 100 were investigated by tensile testing, see Figure 5 and Table 1. The original HEC exhibited the highest E‐modulus of 4524 MPa in combination with low elongation at a break of 9% in correlation with the relatively high *T_g_*. Modification of HEC with methyl 6‐aminohexanoate, greatly decreased the modulus and stress at break, while elongation was increased. This was coupled with the 50 °C decrease in *T_g_*. The modulus and tensile stress at break again increased after the crosslinking reaction, while the flexibility as determined by elongation at break was retained in comparison to HEC‐NH_2_. No large differences were observed between SBHEC 50 and SBHEC 100. Interestingly, both SBHEC materials retained their mechanical properties, including modulus, tensile stress, and elongation, well during reprocessing (Figure 5). Previously reported vanillin‐derived polyester‐imines and epoxy thermosets typically demonstrated tensile stress values between 15 and 50 MPa and elongation of 3–10%,^[^ ^16^, ^28^, ^34^ ^]^ although elongation up to 300% has been reported.^[^ ^38^ ^]^ On the other hand, Schiff‐base cellulose materials consisting of dialdehyde cellulose nanofibrils cross‐linked with diamines demonstrated tensile stress between 20 and 93 MPa and elongation of 1–4%.^[^ ^35^, ^39^ ^]^ In comparison our materials are more flexible with higher elongation and lower tensile stress. The observed difference in the tensile properties compared to previous cellulose Schiff bases is likely explained by the type of cellulose used, i.e., chemically modified thermoplastic cellulose in the present study and cellulose nanofibrils in the previous studies.

### 2.4 Chemical Resistance and Closed‐Loop Chemical Recyclability of SBHEC

**Figure 6:**
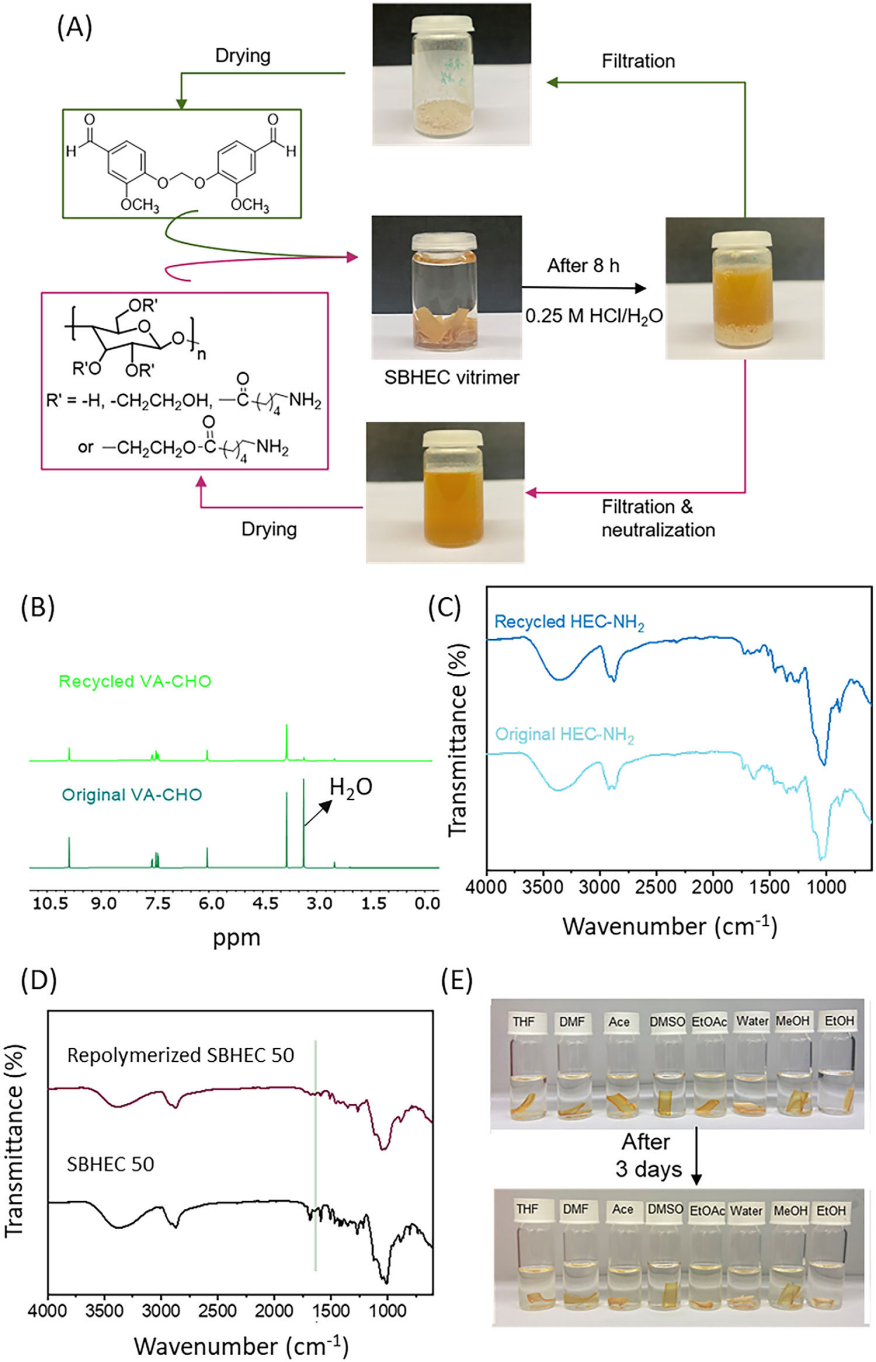
A) Scheme describing the closed‐loop chemical recycling of SBHEC 50 films. B) ^1^H NMR spectra of original and recycled VA‐CHO. FTIR spectra of C) original and recycled HEC‐NH_2_, and D) virgin and re‐polymerized SBHEC 50. E) Solvents resistance of SBHEC 50 films in different solvents.

Based on the known reversibility of the imine bonds under acidic conditions, we developed a facile closed‐loop recycling process by utilizing imine‐hydrolysis at room temperature (**Figure** **6**A). SBHEC 50 was cut into pieces and placed in 0.25 m HCl. The samples were stirred at room temperature for eight h to recycle SBHEC 50 back to the original building blocks VA‐CHO and HEC‐NH_2_ by utilizing imine hydrolysis. The formed HEC‐NH_2_ in the form of HEC‐NH_3_^+^ spontaneously dissolved in the acidic water, while VA‐CHO precipitated as a solid. The VA‐CHO was filtered and washed with water and EtOH several times and dried in the oven at 60 °C. The HEC‐NH_2_∙HCl was converted into HEC‐NH_2_ through base treatment (KOH/MeOH) and distillation. Reformation of HEC‐NH_2_ and VA‐CHO was supported by ^1^H NMR and FTIR spectra. In the ^1^H NMR spectra of recycled VA‐CHO the aldehyde functionality is clearly visible at δ 9.80 ppm and the spectrum is basically identical to the spectrum of the originally synthesized VA‐CHO. 81% of the original VA‐CHO was recovered (Figure 6B). The FTIR spectrum of recovered HEC‐NH_2_ in Figure 6C is also similar to the spectrum of as‐synthesized HEC‐NH_2_. The recovered HEC‐NH_2_ and VA‐CHO were then utilized for a new Schiff base reaction between the aldehyde groups in VA‐CHO and amine groups in HEC‐NH_2_. As shown in Figure 6D, the re‐polymerized SBHEC 50 also exhibits a very similar FTIR spectrum to the virgin SBHEC 50. HEC‐NH_2_ was completely water soluble (Figure S4, Supporting Information), while SBHEC 50 demonstrated good water resistance, a vital ability for a wide range of packaging and other applications. This decrease in solubility further supports the successful Schiff base reaction and formation of cross‐linked structures. After soaking in various solvents for three days at room temperature (Figure 6E), almost no mass loss was observed for SBHEC 50. The results demonstrate that SBHEC 50 has good resistance to water and various solvents, such as ethanol, acetone, DMSO, DMF, DCM, THF, and EtOAc. The introduction of dynamic covalent bonds endows SBHEC with the ability to be thermally reprocessed, while at the same time, the material possesses high solvent resistance.

### 2.5 Shear Adhesion Test

**Figure 7:**
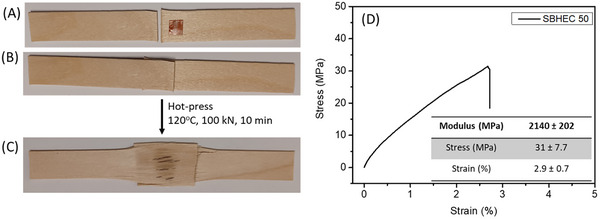
A) Initial wooden pieces with SBHEC 50 B) sandwiched pieces C) after hot‐pressing and D) tensile testing curve.

The potential of the divanillin cross‐linked cellulose material as the adhesive was evaluated by a shear adhesion test, where 50 mg of SBHEC 50 film was placed between two sandwiched wooden blocks that were subsequently hot pressed. The adhesion was then evaluated by tensile testing **Figure** **7**A–D. The test illustrated good adhesion of SBHEC 50 to wood.

## 3 Conclusion

Dynamic cellulose networks were successfully designed by Schiff base reaction between amino‐functionalized 2‐hydroxyethyl cellulose and vanillin dimer. The novel SBHEC materials displayed high thermal stability and solvent resistance, including stability in neutral pH water, opposite to water‐soluble HEC and HEC‐NH_2_. Notably, the synthesized SBHEC could be thermally reprocessed by hot pressing without significant loss of tensile properties. Additionally, the SBHEC could also be chemically recycled through imine hydrolysis at room temperature, enabling the recovery of the original building blocks (HEC‐NH_2_ and VA‐CHO), which could be recured to new SBHEC. Thus, closed‐loop recycling via both chemical and thermal/mechanical routes was realized. The first evaluation of adhesive properties showed good adhesion to wood. This represents a step forward in designing and synthesizing cellulose‐derived dynamic networks integrating biobased raw materials and circularity.

## 4 Experimental Section

### Materials and Chemicals

2‐hydroxyethyl cellulose (HEC, *M_n_* 37 400 g mol^−1^, *M_w_* = 100 900 g mol^−1ṅ^ and Ð = 2.7 as determined by size exclusion chromatography (SEC) in DMSO. Calibration with narrow dispersity Pullulan standards (PSS, Germany)), D‐(+)‐glucose (≥ 99.5%), methyl 6‐aminohexanoate hydrochloride (≥ 99%), dibutyltin (IV) oxide (DBTO, 98%), vanillin (VA, 99%), dibromo methane (99%), potassium carbonate (K_2_CO_3_, ≥ 99%), potassium iodide (KI, ≥ 99%), potassium hydroxide (KOH), hydrochloric acid (HCl) were obtained from Sigma–Aldrich. Dimethyl sulfoxide (DMSO, spectrophotometric grade, 99.9+%) was purchased from CHEMSOLUTE. Acetonitrile (CH_3_CN, ≥ 99.9%), dichloromethane (DCM), tetrahydrofuran (THF), *N, N*‐dimethylformamide (DMF) were obtained from Thermo Fisher Scientific. Ethyl acetate (EtOAc), ethanol (EtOH), acetone, and methanol (MeOH) were purchased from VWR Products LLC. All the starting reagents and solvents for the synthesis were used without further purification.

### Synthetic Procedures—Synthesis of Vanillin Dimer

The vanillin dimer (VA‐CHO) was synthesized by reacting vanillin with dibromomethane. In brief, vanillin (17.44 g, 2 eq.), potassium carbonate (17.28 g, 2.2 eq.), and potassium iodide (0.054 g, 0.1 eq.) were sequentially added into acetonitrile (50 mL). Then, dibromomethane (4 mL, 1 eq.) was added to the mixture and refluxed at 80 °C for 24 h under a nitrogen atmosphere. After the reaction, the mixture was poured into ice water to form a white residue, and the precipitate was collected by vacuum filtration and dried at 30 °C in a vacuum oven to obtain VA‐CHO.

### Synthetic Procedures—Synthesis of Amine‐Functionalized HEC (HEC‐NH_2_)

HEC (1.0 g), methyl 6‐aminohexanoate hydrochloride (0.5 g, 2.5 mmol), and dibutyltin (IV) oxide (10 mg, 0.04 mmol) were added to DMSO and the mixture was reacted at 60 °C for eight h. Next, the solution was poured into 2‐propanol to precipitate the product. The precipitate was collected by filtration and dried at 60 °C in a vacuum oven to get amine‐functionalized HEC. The resulting product was named HEC‐NH_2_.

### Synthetic Procedures—Preparation of Schiff Base HEC (SBHEC)

Two different SBHEC were prepared by Schiff base reaction between HEC‐NH_2_ and different amounts of VA‐CHO. First, HEC‐NH_2_ (1.0 g) was added into 10 mL DMSO: EtOH (1:1) and stirred at 80 °C for two h to dissolve properly. Then different amounts of VA‐CHO (0.5 or 1.0 g) were added and stirred at 60 °C for eight h. Finally, the solution was poured into acetone to form a precipitate. The precipitate was collected by filtration and dried at 60 °C in a vacuum oven. The samples were named SBHEC 50 and SBHEC 100 based on the amount of VA‐CHO (wt.% of HEC‐NH_2_) used in the synthesis.

### Synthetic Procedures—Fabrication and Mechanical Recycling of SBHEC Films

The different SBHEC powders were uniformly placed on a flat plate with 3 cm × 5 cm dimensions and hot pressed at 120 °C, 0.1 MPa for 30 min to obtain the corresponding SBHEC films. For mechanical recycling SBHEC films were further cut into small pieces and subjected to a new hot‐pressing cycle at 120 °C, 0.1 MPa for 30 min to get mechanically recycled SBHEC 50 MR and SBHEC 100 MR.

### Characterization

The chemical structures of the intermediate and final products were characterized by ^1^H nuclear magnetic resonance (NMR) (400 MHz, DMSO‐*d_6_*) spectroscopy using a Bruker Avance‐III 400 MHz spectrometer (Switzerland) and Fourier transform infrared (FTIR) spectroscopy (Perkin‐Elmer, Spectrum 100 FT‐IR Spectrometer) over the range of 600–4000 cm^−1^. The thermal stability of the materials was evaluated by thermogravimetric analysis (TGA) Mettler Toledo TGA 1 under the N_2_ atmosphere with a purge rate of 50 mL min^−1^ and a heating rate of 10 °C min^−1^ from 30 to 600°C. The glass transition temperatures (*T_g_*) were recorded by a TA Q800 dynamic mechanical analysis (DMA) machine (USA) in tension mode in the temperature range 30–300°C. The heating rate and test frequency were 5 °C min^−1^ and 1 Hz, respectively. The mechanical properties of the SBHEC films were investigated by carrying out tensile testing on an Instron 5565 universal mechanical testing instrument (USA) mounted with a maximum 0.5 kN detection cell. The size of the specimens for the tensile test was 50 mm × 5 mm × 2 mm. The clamp distance and tensile rate were 20 and 5 mm min^−1^, respectively. The tensile measurements were repeated with at least three independent samples.

### Solvent Resistance and Chemical Recycling

The solvent resistance of SBHEC films was evaluated by immersing pieces of the films (50 mg) in various solvents (2 mL), including DMF, DMSO, acetone, EtOAc, EtOH, DCM, THF, MeOH, and water (H_2_O) for 3 days at room temperature. To evaluate the chemical recyclability, SBHEC 50 specimens (2 g) were cut into pieces and added to 0.25 m HCl/H_2_O (50 mL). The samples were stirred at room temperature for eight h to recycle SBHEC back to the original building blocks VA‐CHO and HEC‐NH_2_. The released HEC‐NH_2_ in the form of HEC‐NH_3_^+^ spontaneously dissolved in the acidic water, while VA‐CHO precipitated as a solid. The VA‐CHO was filtered and washed with water and EtOH several times and dried in the oven at 60 °C. HEC‐NH_2_∙HCl was converted into HEC‐NH_2_ through base treatment (KOH/MeOH) and distillation.

### Shear Adhesion Test

A shear adhesion test was performed by placing 50 mg of SBHEC 50 film between two rectangular (sandwiched) wooden blocks 50 mm in length, 15 mm in width, and ≈1.0 mm thickness, and placing them in a hot‐press at 120 °C and 100 kN for 10 min. Afterward the shear adhesion was evaluated by a universal tensile testing machine. The sample size was a length of 65 mm, width of 15 mm, and thickness of ≈1.0 mm. 2 kN load cell, and speed 0.1 mm min^−1^ were used.

## Conflict of Interest

The authors declare no conflict of interest.

## Supporting information



Supporting Information

## Data Availability

The data that support the findings of this study are available from the corresponding author upon reasonable request.
