# Supplementary material for: Divanillin Cross‐Linked Recyclable Cellulose Networks
Source: Macromol Rapid Commun. 2025 Mar 26;46(12):2401094. doi: 10.1002/marc.202401094 (PMC12183153; doi:10.1002/marc.202401094)
Supplement: Supplementary file 1 — Supporting Information [file MARC-46-2401094-s001.docx]

**Supporting Information**

# **Divanillin Crosslinked Recyclable Cellulose Networks**

## *Meiling Zhang^a,b#^, Sathiyaraj Subramaniyan^a,c#^, Minna Hakkarainen ^a,c*^*

^a^KTH Royal Institute of Technology, Department of Fibre and Polymer Technology, 10044 Stockholm, Sweden

## ^b^Taiyuan University of Technology, College of Textile Engineering, Jinzhong 030600, Shanxi, China

## ^c^KTH Royal Institute of Technology, Wallenberg Wood Science Center (WWSC), 10044 Stockholm, Sweden

#equal contribution

*Corresponding author: [minna@kth.se](mailto:minna@kth.se)

**List of content**

**Scheme S1.** Synthesis of amino-modified glucose.

**Figure S1.** ^1^H NMR spectra of glucose and amine-functionalize glucose.

**Figure S2.** (A) TGA weight loss and (B) derivative curve of VA-CHO.

**Figure S3**. (A and B) FTIR spectra of original and mechanically recycled HEC-NH2, SBHEC 10 and SBHEC 50.

**Figure S4.** Opposite to SBHEC materials, HEC-NH_2_ dissolved in water.

This supporting information contains 1 Scheme and 4 Figures on 3 pages.

**Scheme S1.** Synthesis of amino-modified Glucose.


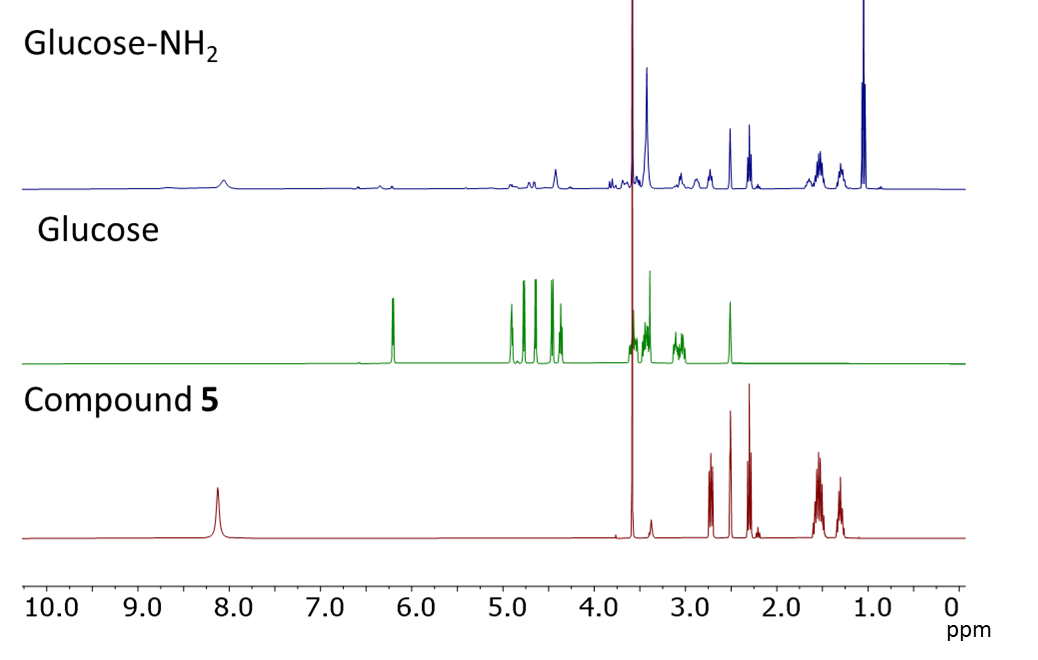


**Figure S1.** ^1^H NMR spectra of glucose and amine-functionalize glucose.


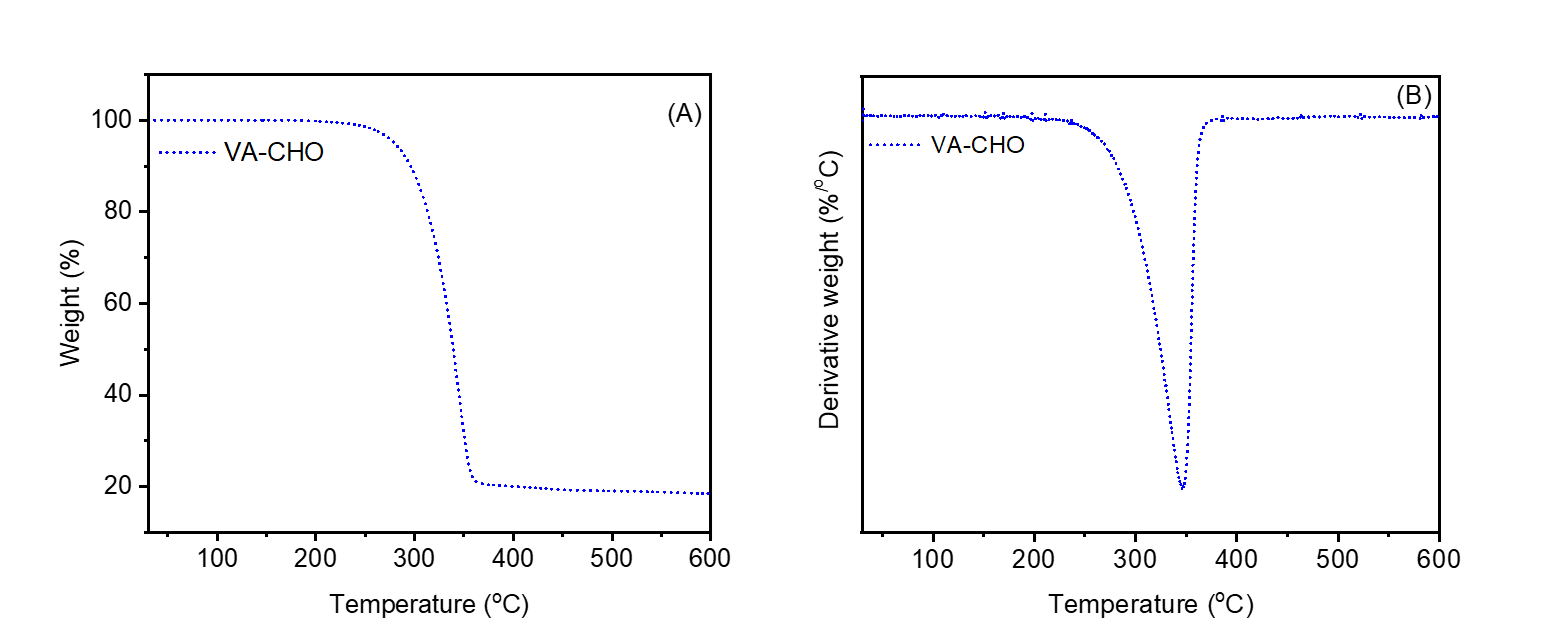


**Figure S2.** (A) TGA weight loss and (B) derivative curve of VA-CHO.


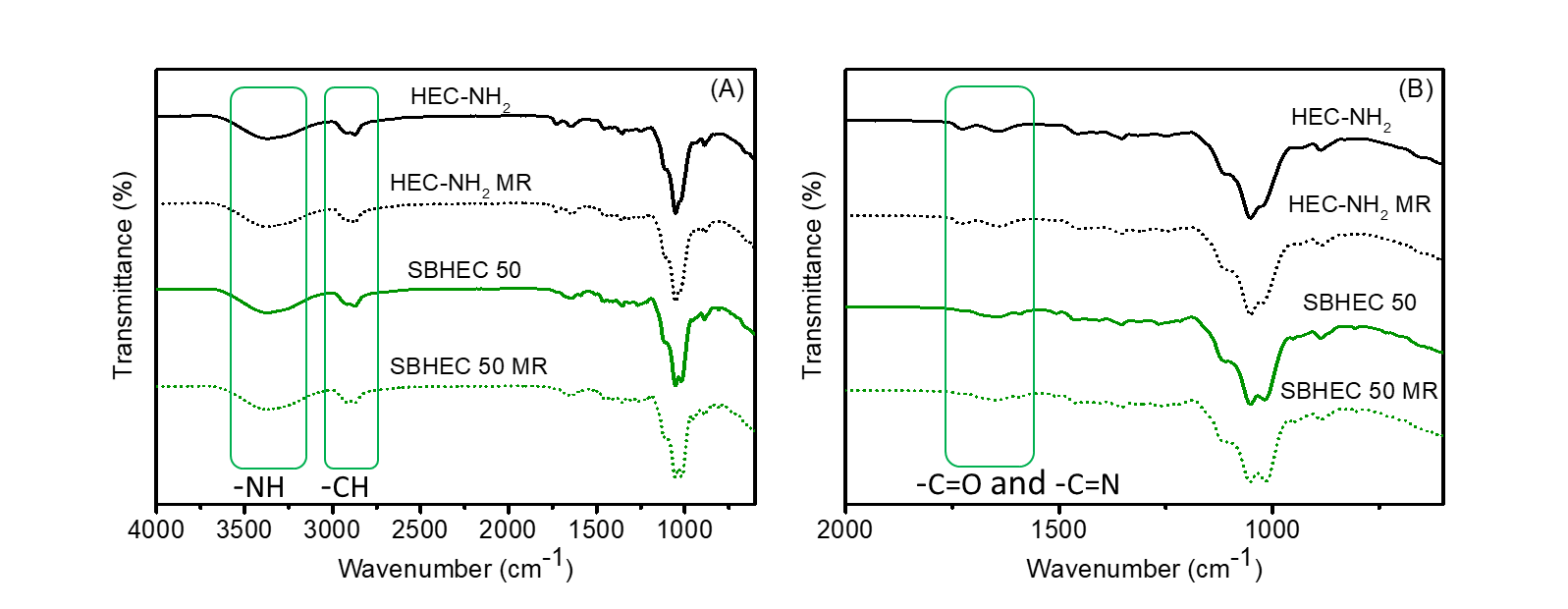


**Figure S3**. (A and B) FTIR spectra of original and mechanically recycled HEC-NH_2_ and SBHEC 50.


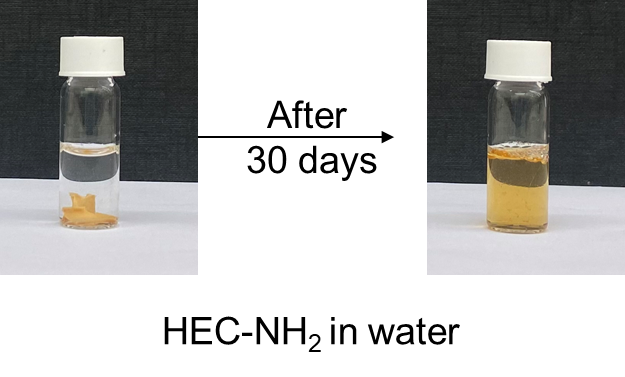


**Figure S4.** Opposite to SBHEC materials, HEC-NH_2_ dissolved in water.
